# Vertical and diel niches modulate thermal selection by rainforest frogs

**DOI:** 10.1098/rspb.2024.1497

**Published:** 2024-11-13

**Authors:** David H. Klinges, Tsitohaina Randriambololona, Zachary K. Lange, Julia Laterza-Barbosa, Herizo Randrianandrasana, Brett R. Scheffers

**Affiliations:** ^1^ School of Natural Resources and Environment, University of Florida, 2035 McCarty Hall D, Gainesville, FL 32611, USA; ^2^ Département de Biologie Animale, University of Antananarivo, Antananarivo, Madagascar; ^3^ Madagascar National Parks, BP 1424 Ambatobe, 103, Antananarivo 103, BP 1424, Madagascar; ^4^ Department of Biology, University of Texas at Arlington, 501 S. Nedderman Dr, Arlington TX 76019, USA; ^5^ Department of Ecology & Evolutionary Biology, Yale University, 165 Prospect St, New Haven CT 06511, USA; ^6^ Départment de Science de la Vie, University of Fianarantsoa, BP 1264 Andrainjato, Fianarantsoa 301, Madagascar; ^7^ Wildlife Ecology and Conservation, University of Florida, 110 Newins-Ziegler Hall PO, Gainesville, FL 32611, USA

**Keywords:** amphibian, arboreal, Madagascar, thermoregulation, thermal preference, tropics

## Abstract

Thermoregulatory behaviour determines an organism’s body temperature and therefore its physiological condition, and may differ for organisms situated across climate gradients. Species’ preferred or selected temperatures may be higher in warmer locations—referred to as coadaptation—or lower in warmer temperatures—countergradient variation. Here, we tested if rainforest amphibians exhibited coadaptation or countergradient thermal selection across an underappreciated spatial climate gradient (vertical height from forest floor to canopy) and separating diel activity (diurnal versus nocturnal behaviour). We captured 2534 amphibians over 216 ground-to-canopy surveys, and conducted 282 thermal selection assays for 37 species while pairing microclimate measurements and mechanistic model predictions to understand vertical and daily thermal variation in the field. Amphibians exhibited countergradient thermal selection: species occupying cool nocturnal conditions in canopies selected warmer temperatures than species occupying hot diurnal conditions at the forest floor. Furthermore, amphibians selected warmer temperatures than the average conditions that they were exposed to when active, and this divergence was especially high for nocturnal arboreal species (8.68°C). This suggests that rainforest amphibians dramatically underfill the warm end of their thermal niches, a trend across local thermal gradients that reflects recent findings across elevational and latitudinal gradients. We show that considering multidimensional climate gradients is important to evaluate thermoregulatory behaviour, and its evolutionary underpinnings, for understanding species’ niches and community assembly.

## 1. Introduction

Thermoregulatory behaviour determines an organism’s exposure to climate variation, which influences its biological function and fitness [1]. For ectotherms, body temperatures closely correspond to environmental temperatures, making the temperatures they select particularly important in determining physiological function. Thus, especially in ectotherms, thermoregulation acts as a natural link between organismal behaviour and physiology [2]. Temperatures selected by organisms have been observed to vary intra- and interspecifically across environments [1–3], which influences ecology and potential species’ vulnerability to contemporary climate change [4]. Here we use the term ‘thermal selection’ (Tsel), which is not linked to a given *a priori* behavioural mechanism, rather than ‘thermal preference’, which assumes a thermotactic behavioural drive [5].

**Figure 1.**
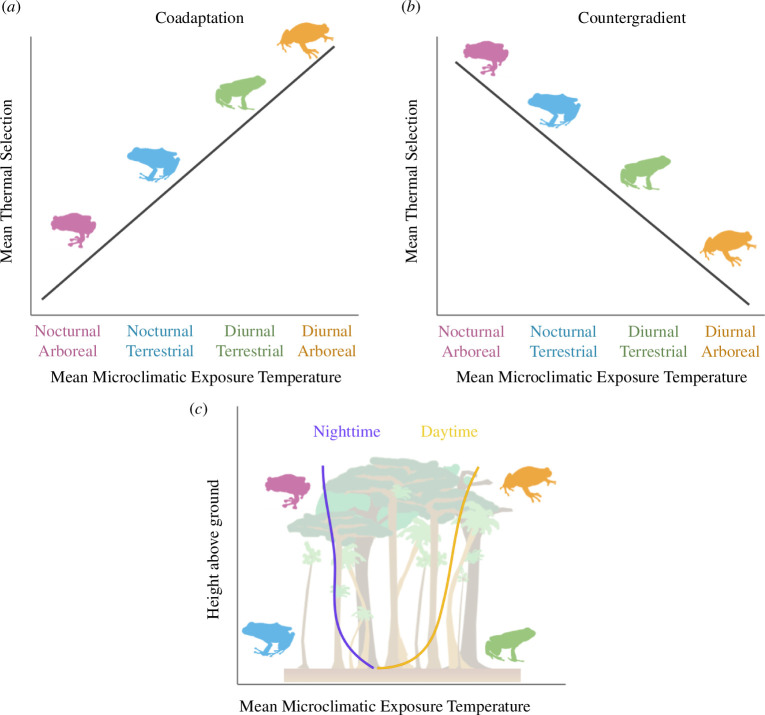
Two predominant hypotheses, coadaptation and countergradient variation, may explain how thermal selection differs for thermoregulatory organisms across an environmental gradient. Coadaptation (*a*) posits that thermal selection will be positively correlated with mean environmental temperatures, as the thermal niche is attuned to the most common temperatures. Countergradient variation (*b*), however, suggests a negative relationship, whereby organisms select for conditions that are physiologically beneficial, yet rare, within their environment. In the context of forested systems (*c*), in which nighttime and daytime temperatures express vertical gradients (purple and yellow lines), coadaptation would entail that nocturnal arboreal (pink) species would have the coldest selected temperatures, given their exposure to the coolest conditions, and diurnal arboreal (orange) species would have the warmest selected temperatures. Countergradient variation would predict the opposite: nocturnal arboreal species must compensate for their cool environment by seeking out warmer temperatures and diurnal arboreal species select for cooler temperatures.

Two leading hypotheses, coadaptation and countergradient variation, have been proposed to explain how intraspecific or interspecific variation in thermal niches, including Tsel, relate to environmental gradients (figure 1). We acknowledge that while the usage and ramifications of each hypothesis in ecology are broad, here we explore them primarily as they relate to thermoregulation. The coadaptation hypothesis emphasizes that Tsel tends to be linked to thermal optima, and both Tsel and thermal optima track environmental temperatures [6,7]. Thus, coadaptation intuitively predicts that Tsel should positively correlate with average environmental temperatures, such that performance is optimized to the conditions that are most common [6,8]. The coadaptation hypothesis also relies on the assumption of trade-offs between warm and cool adaptation [9], in that specializing for higher performance under warmer temperatures entails lower performance under cooler temperatures, and *vice versa* [2,10].

An alternative well-supported hypothesis is of countergradient variation [11–14]. The countergradient hypothesis posits that thermal phenotypes or behaviour negatively correlate with an environmental gradient, i.e. individuals from cooler environments select warmer temperatures than individuals from warmer environments (figure 1). This may occur when organisms seek out conditions that are less common in their environment, yet are physiologically beneficial [15].

Recent examinations of both hypotheses have focused on the spatial variation in Tsel of populations or species, from broad scales such as animals situated across latitude [16] or at the scale of competing species within the same landscape [10]. Such spatial work has also shown that Tsel variation can occur even across microgeography—geographic gradients within the dispersal neighbourhood of a focal organism [17]. Furthermore, a recent reinvigoration of microclimate studies in ecology has shown that many biologically important thermal gradients occur across centimetres to metres [18]. For example, the floor and canopy of forests can have dramatically different thermal regimes, as forest floors typically experience less thermal variability due to the buffering effect of vegetation above [19]. Thermal buffering from vegetation can thus reduce the exposure of forest organisms to physiologically intolerable conditions [20]. In fact, differences in thermal regimes across vertitude—that being the vertical axis from forest floors to canopies—are comparable to thermal differences expressed across hundreds of metres in altitude, or thousands of kilometres in latitude [21]. The vertical thermal gradient has been attributed to generating vertical community structure and turnover between arboreal (canopy-dwelling) and terrestrial species [22] and is a prominent driver of diversification [23].

Differences in thermal regimes across vertitude are due to the magnitude of temperature fluctuations between daytime heat and nighttime cold, both frequently more extreme in the forest canopy than at the floor [19]. If environmental temperatures influence thermal selection, then spatial and temporal gradients may interact to jointly influence Tsel within or across species (figure 1). While some efforts have explored whether Tsel changes across time [24], few studies have explored differences in Tsel across many species that occupy adjacent, yet distinct, spatial and temporal niches (e.g. forest canopy/floor and day/night). Understanding variation in Tsel, especially relative to the temperatures species are exposed to, can help us understand what conditions are optimal for species (but see [1,25]), what biotic and abiotic factors may constrain them from achieving optimal temperatures, and how different thermoregulatory behaviours may be adaptive or maladaptive under contemporary climate change.

Here, we extensively surveyed amphibian communities using day and night, ground-to-canopy surveys and characterized variation in Tsel across a highly speciose rainforest amphibian community in Madagascar. To place Tsel in the context of relevant environmental conditions, we measured and modelled microclimate temperatures from forest floor to canopy. We explore whether patterns of Tsel variation across vertical and diel niches are consistent with the coadaptation hypothesis or countergradient variation. Coadaptation is supported if Tsel positively correlates with a mean microclimatic temperature of a species’ spatiotemporal niche, whereas countergradient is supported if Tsel negatively correlates with microclimatic temperature. Furthermore, we predict that a species’ niche in space (vertical occupancy) and time (diel activity) will in tandem better predict Tsel than either component of scale considered by itself.

As ectotherms, amphibians are highly sensitive to environmental temperatures [2], and the unique morphological adaptations for jumping or climbing entail that many species are specialized for life on the ground or in the trees, but rarely both simultaneously [26]. Furthermore, Malagasy amphibian species include both diurnal and nocturnal species, which experience dramatically different temperatures during activity [27]. We observed that arboreal amphibians exhibited countergradient variation in Tsel across time, and that diurnal amphibians exhibited countergradient variation in Tsel across space. In addition, we find that Malagasy amphibians, especially nocturnal arboreal species, underfill their thermal niche on the warm end due to daytime inactivity. We discuss possible underlying mechanisms that could give rise to such patterns and the implications of local deviation in thermal selection for ecophysiology and niche partitioning.

## 2. Methods

### (a) Field sampling and trait measurements

**Figure 2.**
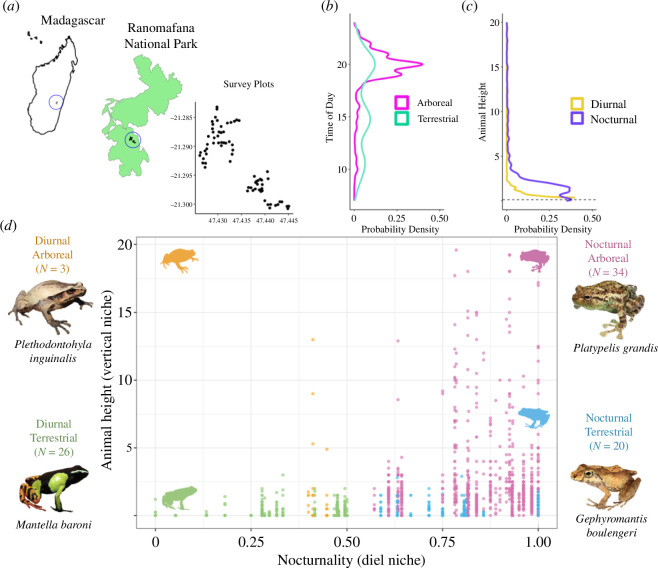
Community assembly of rainforest amphibians across space (vertical occupancy from forest floor to canopy) and time (diel cycle). (*a*) Map of vertical amphibian survey locations (*n* = 73) in Ranomafana National Park, Madagascar. (*b*) Most amphibians were encountered at night (time axis displayed here is truncated to remove times of low sampling between 00.00 and 07.00 h). (*c*) Nocturnal species typically were observed higher in the canopy than diurnal species. (*d*) The distributions of vertical and diel niches of amphibians surveyed (83 species). Each point represents one individual, coloured according to four categories of vertical and diel niches, each category also displayed with an image of a representative species and the number of species encountered.

We surveyed an anuran (frogs and toads) assemblage in the montane broad-leaf rainforest of Ranomafana National Park, Southeastern Madagascar (21°17′ S, 47°25′ E; figure 2). Our sampling areas (Vatoharanana and Valohoaka) represented a narrow elevational range of 969–1171 m [28]. Over two 3-month periods from 2018 to 2020 (November 2018–January 2019; November 2019–January 2020), we conducted vertical ground-to-canopy surveys for amphibians. Using survey methods from [29], each survey was centred on one of 73 randomly selected trees that met safety standards for arborist single-rope climbing [30]. To sample both diurnal and nocturnal species, we performed at least one daytime (08.00–16.00) and one nighttime (20.00–02.00) climb per tree per year (total of 216 surveys). We alternated the first survey, day or night, to avoid temporal biases in sampling. We recorded the maximum survey height climbed and tree height using a laser distance recorder (Leica Disto D2; http://www.leica-geosystems.com).

At each tree, we conducted a 60-min survey from ground to canopy, conducting ground surveys first to avoid biases caused by amphibians jumping from the trees to the ground during arboreal surveillance. We confined ground surveys to a 4-m radius surrounding the bottom of the climbing rope, corresponding to the aerial area sampled by a climber attached to the rope. During ground surveys, we searched leaf litter, logs and other amphibian microhabitats [31]. During arboreal surveys, we searched tree holes, epiphytes and other microhabitat structures. We recorded the height of each amphibian captured, and for downstream analyses represented this capture height as a proportion of the maximum height of the canopy (henceforth ‘relative height’). Given the variation in tree canopy heights in this study (15.8–35 m), relative height serves as a better proxy than the absolute height of a small animal’s exposure to above-canopy downwelling radiation, and therefore its vertical niche as relevant to thermal exposure.

Upon capture, we identified each animal to species using a field guide to Malagasy amphibians [32] and measured each animal’s mass, which has a demonstrated link to Tsel [33,34]. For quantifying the diel niche, each species was also provided with a nocturnality score derived from the proportion of all individuals of that species that were captured after sunset and before sunrise (0 = no individuals; 1 = all individuals; figure 2). This nocturnality score better reflects species-level activity patterns than the exact time of capture for each individual, as some predominantly diurnal species were captured by chance encounter at night (e.g. a sleeping frog startled from hiding) and *vice versa*. The nocturnality score was used to define the diel niche in statistical modelling (see §2d). To communicate findings separately for diurnal and nocturnal species, we also designated a species categorically as nocturnal if its nocturnality score was above 0.5 (i.e. over 50% of individuals of that species were found at night), and diurnal otherwise. As only 16.9% of species had nocturnality scores between 0.4 and 0.6, it was intuitive to classify species as either nocturnal or diurnal [35]. Each species was also categorically designated as terrestrial or arboreal as defined in the AmphiBIO database [36], which aligned with our observations of capture heights and behaviours.

### (b) Temperature monitoring and modelling

To estimate vertical environmental temperatures from forest floor to canopy, we modelled forest temperatures using the *microclimc* microclimate model [37]. In brief, *microclimc* estimates the transfer and absorption of heat and vapour through vegetation canopies following first principles [19] to predict air, leaf and soil temperature, and relative humidity, drawing upon *NicheMapR* [38] for rapid calculation of soil moisture and temperature. We used ERA5 hourly data processed via *mcera5* [39] to drive *microclimc*. We parametrized the model using empirical canopy heights, the model’s built-in vegetation parameters for evergreen broadleaf forests, and soil parameters via *microclimc::soilinit*(). We ran the model for the year 2019 (when most amphibians were collected) corresponding to the approximate locations of nine temperature loggers deployed at multiple heights within the forest. Loggers were placed 0.1–17 m aboveground on trees within the study landscape (electronic supplementary material, table S1). Temperature sensors were deployed in locations likely occupied by frogs, including on branches, tree holes and spaces between roots (electronic supplementary material, figure S1). Sensors exposed to direct sunlight were placed within a PVC pipe to shield them from radiation, although such shielding limits sampling of high temperatures. Loggers recorded microclimate for periods between 2 and 12 months.

We modelled microclimate at 20 vertical heights between 0 and 25.4 m, which we validated using 2019 logger measurements, recording the root mean square error (RMSE) in model predictions at monthly resolution. For analyses presented in the main text, we used spatially averaged model predictions of leaf temperatures, rather than air temperatures, as they more closely represent the boundary-layer conditions that small-bodied amphibians experience while perched on leaves [34,40]. Use of air temperatures in analyses did not qualitatively change results or interpretations (see §3).

### (c) Thermal selection experiments

We determined amphibian Tsel experimentally, rather than from field body temperatures, as laboratory data reflect selected temperatures without costs or constraints imposed in field conditions [41]. We used gradient-based Tsel arenas reflecting prior studies [42,43]. We constructed five enclosed arenas using aluminium downspout gutters (150 × 8 × 5 cm), each covered with a hard plastic lid (electronic supplementary material, figure S2). The warm end of the arena rested on two strips of heat tape, and the cool end rested on frozen gel packs. Heat tape was activated 1 h before the trial started to allow temperatures to equalize. We used heat tape rather than heat lamps because lamps that emit light can have adverse effects on measuring Tsel of nocturnal species [24]. This generated a thermal gradient of approx. 10–36°C, representative of thermal conditions in Ranomafana National Park (see §3). The arena’s interior was lined with moist paper towel substrate to prevent amphibian desiccation. Paper towels were re-misted if not visibly wet.

Amphibians underwent thermal selection trials within 48 h of capture, during which they were acclimated at approx. 22°C in ventilated moist, and shaded containers. As animal thermal selection may vary with the time of day or sunlight exposure [44,45], all trials were conducted only in afternoons and evenings (between 15.45 and 21.54) in a laboratory with covered windows and lit only by overhead lights. For each trial, one animal was gently placed in the arena’s middle with the lid then closed. To measure temperatures, we partially opened the arena lid (to minimize airflow) to record the animal’s dorsal surface temperature, and the arena substrate temperature adjacent to the animal, using an infrared thermometer (Etekcity^®^). We used a handheld weather meter (KestrelMeters^®^) to confirm relative humidity was consistently about 80%. In the first year of assays, we measured temperature and humidity every hour across each 4-h trial, while in the second year, we measured every 15 min; we accounted for such interannual methodological differences in downstream modelling (see §2d). Trials continued for 4 h unless the animal was in critical condition and removed (*n* = 5; not included in analyses). Given that arboreal amphibians may instinctively seek to move vertically rather than horizontally, we noted whether an animal was attached to the arena walls or lid. Furthermore, as amphibians may undergo desiccation stress even despite our moist paper towel substrate, we monitored when each animal was in a water conservation posture: limbs tucked close to the body to reduce surface area [40]. At the end of each trial, paper towels were disposed of and all materials were wiped with ethanol to remove any waste or pheromones. Amphibians were released at their capture location within 48 h after experimentation. All animal handling and processing was performed according to IACUC Study 201709756 and approved by the University of Florida.

### (d) Analysis

For each animal, we averaged its body temperature measurements across the 4-h trial period, and calculated the s.d. in Tsel across the trial. Given that animals may initially explore their environment rather than immediately exhibiting a selected temperature, we also conducted downstream analyses removing the first 2 h of trials as done in prior studies [46,47].

We fitted generalized linear models (GLMs) with individual amphibian mean Tsel as the response variable, normal error structure and morphology (mass) and niche components (diurnal/nocturnal, arboreal/terrestrial) as predictors. Prior to fitting models, all covariates were scaled between 0 and 1 to standardize contributions of each parameter. We first fit three GLMs: (i) a ‘temporally naive’ model (i.e. no temporal predictors) with an animal’s mass and the relative height at which it was found as predictors; (ii) ‘spatially naive’ model (i.e. no spatial predictors) with mass and nocturnality; and (iii) a ‘spatiotemporal’ model with mass, relative height and nocturnality, with an interaction term between relative height and nocturnality. This set of models allowed us to explore the separate and combined effects of an animal’s capture height (spatial niche) and nocturnality (temporal niche) on its mean Tsel. To further explore patterns of Tsel separately for categorical subsets of the amphibian assemblage (e.g. for just nocturnal animals, how important is relative height for Tsel?), we fit a temporally naive model from only nocturnal animals, and from only diurnal animals; we also fit a spatially naive model only from arboreal animals, and separately only from terrestrial animals. Given that the variation in Tsel may also differ for animals occupying separate vertical and diel niche spaces, we also fit temporally naive, spatially naive and spatiotemporal models of the same structure as above, but with the s.d. in Tsel as the response variable rather than mean Tsel. For these models, we fit GLMs with a Gaussian log link function for the response, given positive skew and non-negative values for Tsel s.d.

We tested for correlations between multivariate predictors using Spearman’s *ρ*, given the non-normality of predictor distributions, and calculated variance inflation factors (VIFs) for all covariates to quantify multicollinearity [48]. We also fit models with sampling period (2018–2019; 2019–2020) as a random effect to control for interannual variation in environmental conditions and methodology.

We tested for the strength of phylogenetic signal on both the mean and s.d. of Tsel. While the phylogenetic relationships of Malagasy amphibians are actively researched [49], our study includes primarily well-studied species. We used 100 trees drawn randomly from [50], from which we calculated the median tree (i.e. the single binary tree that represents the geometric median of the 100 tree topologies) [51]. Using this median tree, we obtained the phylogenetic signal of Tsel as measured by the *K* statistic and Pagel’s lambda values, using the *phylosig* function of the *phytools* R package [52]. Although we found only a weak phylogenetic signal of mean Tsel as measured by Pagel’s lambda (*λ* < 0.001) relative to other traits such as nocturnality (*λ* = 0.52; Pagel’s lambda varies from 0 to 1), we nevertheless fitted phylogenetic GLMs as they do not erode performance relative to non-phylogenetic GLMs [53], using the R function *caper::pgls* [54]. Given the weak phylogenetic signal of Tsel, we did not use taxonomic information as random effects in multivariate models, but rather used functional traits (e.g. mass, nocturnality) to explain thermal niche variation, for which there were more clear ecological hypotheses.

To examine whether amphibian Tsels exhibited coadaptive or countergradient patterns relative to environmental temperatures, we associated each animal with an average of the *microclimc* temperature predictions that best represented that animal’s thermal exposure in space and time. To do so, for each individual we selected the *microclimc* hourly predictions corresponding to the height at which the animal was captured. Then, we calculated a weighted average of modelled daytime and nighttime temperatures based on the animal’s nocturnality score. For example, if an amphibian was captured at 3.3-m height, and its species had a nocturnality score of 0.6, *microclimc* predictions for 3.3-m height were averaged across time, with nighttime temperatures given a weight of 0.6, and daytime temperatures a weight of 0.4. This allowed us to derive a single temperature value that adequately represented an animal’s thermal exposure in space and time (mean microclimatic exposure temperature). We then fitted linear regressions of Tsel across mean microclimatic exposure temperature, for all individuals, and arboreal and terrestrial species separately. We used paired Wilcoxon signed rank tests to compare mean Tsel against the predicted mean temperatures at the height at which animals were found, drawn from daytime (for diurnal species) or nighttime (for nocturnal species) model predictions. Finally, as variation in environmental temperatures may relate to the mean or variation in selected temperatures, we fitted linear regressions of mean Tsel across the s.d. of microclimatic exposure temperature, and of s.d. in Tsel across the s.d. of microclimatic exposure temperature.

## 3. Results

From 216 surveys, we captured 2534 individuals representing 83 species of 15 genera (figure 2). Of these animals, 282 individuals of 37 species underwent Tsel experiments. Collectively, animals spent < 10% of the time during trials attached to arena walls or lids, or in a water conservation posture. Per-species Tsel ranged from 19.63°C (*Boophis picturatus*) to 27.66°C (*Mantidactylus majori*), with an average of 24.0°C (electronic supplementary material, table S2).

**Figure 3.**
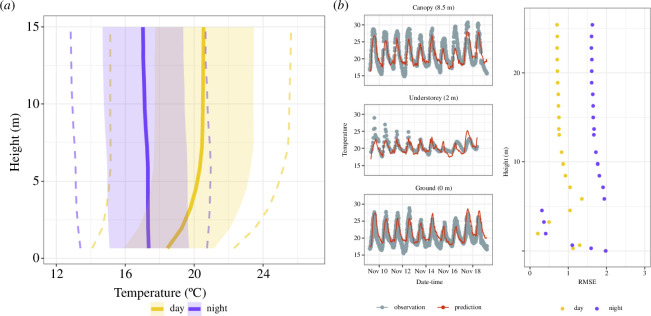
(*a*) Microclimate model-predicted vertical profiles, indicating cooler nighttime temperatures and warmer daytime temperatures, with a more pronounced vertical gradient, and greater thermal variation, during the day. Solid lines indicate mean temperatures across height, shaded bands indicate 25% and 75% quantiles, and dashed lines indicate 10% and 90% quantiles, for day (yellow) and night (purple). (*b*) Model predictions reflected empirical logger measurements across time and space, with low RMSE (°C) in model predictions across all heights.

The *microclimc* model was accurate at capturing forest temperatures, and prediction accuracy did not vary considerably with height or time: average root mean square error (RMSE) was 1.17°C and ranged from 0.21 to 1.97 (figure 3). According to model predictions for the period of field sampling (November–March), temperatures ranged from 13.5 to 27.4°C (2.5% and 97.5% quantiles).

**Figure 4.**
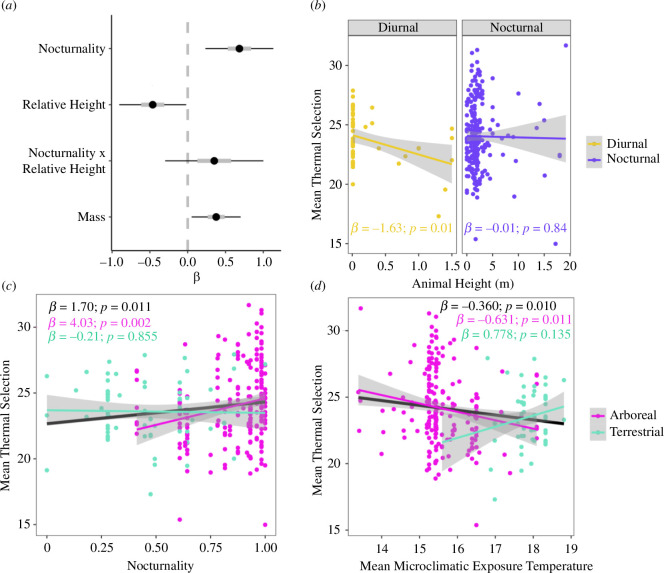
Amphibian selected temperatures diverged according to a species’ temporal and spatial niche. (*a*) Multivariate modelling revealed the strong and counteracting roles of temporal (nocturnality) and spatial (an animal’s relative height) niches. Plotted here are the *ꞵ* coefficient estimates for predictors included in a GLM with individual average thermal selection as the response. Sign of *ꞵ* (±) indicates direction of influence on thermal selection. Black bars show 95% confidence intervals (CIs), grey bars indicate 50% CIs; asterisks indicate predictors for which 95% CIs do not include zero and thus indicate statistically meaningful relationships. Both nocturnality and relative height were more important than morphology (mass). (*b*) Yet, an animal’s relative height was a significant predictor of thermal selection for only diurnal, but not nocturnal, animals (each point in (*b*–*d*) corresponds to one individual; coloured lines indicate mean predictions from linear regressions, grey ribbons indicate 95% CIs of the mean). (*c*) Furthermore, the overall correlation between nocturnality and Tsel (indicated by black text) held for arboreal amphibians (pink text) but not terrestrial species (green text), perhaps given the high thermal separation between daytime and nighttime temperatures in the canopy but not at the forest floor. (*d*) Collectively, there was a significant countergradient pattern of Tsel across the gradient of microclimatic exposure in space and time (black text), and again this pattern was stronger for arboreal species (pink text) and did not hold for terrestrial species (green text).

Of the 37 species that underwent Tsel experiments, 22 were nocturnal-arboreal, six were nocturnal-terrestrial, seven were diurnal-terrestrial and two were diurnal-arboreal. The spatiotemporal GLM (i.e. relative height, nocturnality, mass and relative height × nocturnality as predictors) indicated that both spatial and temporal dimensions were important for Tsel (figure 4, electronic supplementary material, table S3), and was more parsimonious (AIC = 1355.1) than the spatially naive model (AIC = 1361.8) but similar in AIC to the temporally naive model (AIC = 1355.5). Here, coefficient estimates indicated a positive effect of nocturnality on Tsel (i.e. more nocturnal animals selected warmer temperatures; *β* = 0.69, 95% CI = 0.24, 1.13), a negative effect of relative height (i.e. animals found relatively higher selected cooler temperatures; *β* = −0.46, 95% CI = −0.91, −0.02), a positive effect of mass (*β* = 0.38, 95% CI = 0.05, 0.70) and no interaction between nocturnality and relative height (*β* = 0.35, 95% CI = −0.30, 1.00). Given the positive association between mass and Tsel, we also fit a *post-hoc* spatiotemporal model with an interaction term between body mass and nocturnality and found no significant interaction (*β* = 0.21, 95% CI = −0.10, 0.52).

GLMs fit with terrestrial and arboreal species separately revealed that nocturnality was important for arboreal species (*β* = 1.38, 95% CI = 0.71, 2.05), but not for terrestrial species (*β* = −0.067, 95% CI = −0.63, 0.50; electronic supplementary material, table S4); GLMs fit with diurnal and nocturnal species separately revealed that relative height was important for diurnal species (*β* = −3.63, 95% CI = −6.79, −0.46) but not for nocturnal species (*β* = −0.23, 95% CI = −0.60, 0.15; electronic supplementary material, table S4). The spatially naive GLM with Tsel s.d. as the response variable indicated that nocturnality had a significantly positive effect on Tsel s.d. (*β* = 0.13, 95% CI = 0.05, 0.21), although this effect of nocturnality on Tsel s.d. was non-significant in a spatiotemporal GLM (*β* = 0.09, 95% CI = −0.01, 0.20; electronic supplementary material, table S5). Excluding the first 2 h of Tsel trials (electronic supplementary material, tables S6 and S7) and including the sampling period as a random effect (electronic supplementary material, tables S8 and S9) did not qualitatively change any results. Fitting phylogenetic models did not qualitatively change results concerning mean Tsel (electronic supplementary material, table S10), yet phylogenetic models indicated a stronger relationship of mass with the s.d. of Tsel (electronic supplementary material, table S11).

Multivariate model predictors were uncorrelated except for relative height and nocturnality, which had a positive correlation (*ρ* = 0.41, electronic supplementary material, table S12). All covariates in all models had variance inflation factors below 2.3, indicating low multicollinearity [48]. All models met assumptions for linear regressions, including independent predictors and observations, normally distributed residuals and linear relationships between predictors and mean responses [48].

As measured via linear regression, there was a negative correlation between mean Tsel and mean microclimatic exposure temperature (*β* = −0.39, 95% CI = −0.64,−0.087; figure 4). This correlation was significant for arboreal amphibians (*β* = −0.63, 95% CI = −1.12,−0.15) yet non-significant for terrestrial amphibians (*β* = 0.78, 95% CI = −0.25, 1.81; electronic supplementary material, table S13). Furthermore, linear regressions indicated negative correlations of both mean Tsel (*β* = −1.05, 95% CI = −2.02,−0.072; electronic supplementary material, table S14) and Tsel s.d. (*β* = −0.97, 95% CI = −1.46,−0.47; electronic supplementary material, table S15) with the s.d. in microclimatic exposure temperature.

Tsels were skewed warmer than environmental conditions (electronic supplementary material, table S16). On average, individuals selected temperatures that were 7.9°C warmer than the mean temperatures of their observed height and activity period, and this divergence was higher for nocturnal arboreal species (8.68°C)—both differences were statistically significant according to paired Wilcoxon signed rank tests (*W* = 2, *p <* 0.001; *W* = 1, *p <* 0.001).

## 4. Discussion

### (a) Local divergence in thermal selection across space and time

Species that occur across an environmental gradient (e.g. cool to warm conditions) may select temperatures (Tsel) that positively correlate with the gradient—coadaptation—or negatively correlate with the gradient—countergradient variation (figure 1). Here, we explored Tsel for rainforest amphibians occupying different diel niches and occurring across an underappreciated biogeographic axis: vertitude, or height within a vegetated system. We observed that collectively across the assemblage, amphibian Tsel demonstrated countergradient variation (figure 4). Specifically, species occupying cooler environments (e.g. nighttime canopies) selected warmer temperatures than species occupying warmer environments (e.g. daytime forest floor).

While coadaptation has been a paramount tenet to thermal ecology [6], countergradient patterns of Tsel may also be common across taxa and geography [13,14]. Warmer temperatures can be physiologically beneficial for ectotherms occupying cooler conditions, such as at night, and therefore favoured by nocturnal species [41]. Diurnal species, on the other hand, may risk overheating [25] and thus may instead seek out cooler conditions [55]. Yet, in our study of a speciose community, such countergradient Tsel was not uniformly observed for species occupying different spatial niches. The countergradient trend was significant for arboreal amphibians, while there was no significant correlation between Tsel and microclimate temperature for terrestrial species (figure 4*d*). This suggests divergent Tsel patterns within an assemblage, modulated by vertical niches.

The patterns of how Tsel diverged across this amphibian assemblage are best understood when the assemblage is separated into the four quadrants of vertical and diel niches (displayed in figure 2*d*), each of which are exposed to different average temperatures and thermal variation. Daytime and nighttime temperatures diverged dramatically in the canopy, with typically less divergence near the forest floor (although for locations in which the forest floor was exposed to full sun, thermal variability was sometimes higher at the floor relative to the understorey, figure 3*b*). Such broad diel thermal variation within forest canopies [21] may drive Tsel divergence between nocturnal and diurnal animals in the canopy, while lower diel thermal variation at the forest floor results in no such Tsel divergence. Our spatiotemporal GLM also suggested that animals captured higher aboveground selected cooler temperatures than animals found lower in height (figure 4*a*). Given that most aboveground animals were nocturnal (upper right quadrant of figure 2*d*), this result may seem in conflict with the above interpretation that nocturnal animals select warmer temperatures. Yet separately analysing nocturnal and diurnal species showed that relative height was only important for Tsel of diurnal animals, not nocturnal animals (figure 4*b*, electronic supplementary material, table S4). Vertical change in environmental temperature is reduced at night (vertical purple line in figure 3*a*) relative to the day (vertical yellow line), with warmer extreme temperatures in the canopy compared to near the ground (electronic supplementary material, figure S3). Thus, nighttime perch height does not dramatically influence cold exposure, while daytime perch height is important for heat exposure, which may explain the role of height in shaping Tsel for diurnal species.

This highlights the importance of accounting for both the correct microhabitats and periods of activity when exploring thermal biology [27,56]. Although ecologists have long studied the roles of diel niches [35] and vertical niches [20,57] in shaping ecology and physiology, few prior works have jointly considered both. In continued work on physiology, we encourage researchers to sample across both spatial and temporal gradients to help disentangle the roles of space and time in shaping thermal exposure and selection. A relevant disclaimer is that there was no significant interaction between nocturnality and relative height in our spatiotemporal model (electronic supplementary material, table S3). This may be due to low statistical power for this test of an interaction term [58], given the truncated distribution of heights for diurnal animals (few animals jointly representing the diurnal and arboreal parameter space). Nevertheless, we caution that our results support additive, but not necessarily interactive, roles of spatial and temporal factors for shaping amphibian Tsel.

### (b) Thermal niche underfilling: arboreal species inactive during the day

Examining Tsel across species within the context of their thermal exposure can help identify relevant biological and behavioural mechanisms. We found that almost all individual amphibians exhibited Tsel above 21°C (electronic supplementary material, table S2), which is warmer than both mean nighttime and daytime temperatures (figures 3 and 4*d*). In fact, for nocturnal arboreal species, Tsels were on average 8.68°C warmer than the mean temperatures they were exposed to when active, and more closely matched the average daytime conditions in the canopy when sunny (i.e. those daytimes above the 90% quantile of downwelling shortwave radiation, electronic supplementary material, figure S3). Given that Tsel almost certainly falls below heat tolerance thresholds [1], nocturnal arboreal species should thus be able to readily tolerate (and in fact select for) typical daytime canopy temperatures. Yet, we observed almost no arboreal amphibians during the day; only 22 animals were found above 3 m in height during daytime (figure 2). This was likely because arboreal amphibians were diurnally inactive—within tree holes and epiphytic plants, but also oftentimes resting on leaf surfaces [20]—and therefore harder to detect. Instead, arboreal amphibians were active at night, which was cooler than their selected temperatures. Angilletta & Werner [24] also observed activity only at times below preferred temperatures for nocturnal geckos, and Huey *et al*. [27] found that such nighttime gecko activity was cooler than their optimal temperatures. Huey *et al*. [27] proposed this was because geckos evolved warm optimal temperatures, despite cooler nighttime activity, given their need for high thermal tolerance limits. Geckos must tolerate hot daytime temperatures (even if diurnally inactive), and evolving warm tolerance tends to yield correlated evolution of warmer thermal optima, and potentially also Tsel [1]. Yet selected temperatures may also be cooler than physiologically optimal temperatures in order to avoid hot lethal extremes given imperfect thermoregulatory abilities (see content on Jensen’s Inequality, e.g. [25]). The same evolutionary mechanisms may be at play for our studied arboreal amphibians. Thus it appears that arboreal nocturnal species are not capitalizing on metabolically productive warm days (i.e. underfilling their thermal niche at the warm end), by remaining inactive.

Interestingly, this pattern recapitulates well-documented patterns of thermal niche underfilling of animals at their warm latitudinal range edge [59] and altitudinal range edge [60], albeit here on a microgeographic scale. Climate constraints remain a prominent hypothesis for explaining the dominance of nocturnality in amphibians [61], yet here it appears clear that these species can tolerate average daytime temperatures. Our findings therefore are enigmatic—why the empty daytime, arboreal niche? We propose two overlapping hypotheses.

The first is that hydric, rather than thermal, constraints may limit arboreal species to nocturnal behaviour. Due to their porous skin, amphibians require access to standing water or humid conditions [40]. Like many ecosystems, tropical rainforests tend to have lower humidity during the day than at night—especially in the canopy [19]—as confirmed by relative humidity predictions by the *microclimc* microclimate model in this study (77.8% mean relative humidity in the daytime canopy, compared with >95% mean relative humidity for nighttime canopy). This may force amphibians to take refuge in moist canopy microhabitats during the day [20]. While some biophysical modelling has shown higher desiccation tolerance of arboreal than terrestrial amphibians [62], whether this physiological divergence exists across forest amphibians globally, including in Madagascar, is unclear. We suggest that hydric constraints likely play a role in the underfilling of arboreal amphibian thermal niches, but given high humidity in Malagasy rainforests, this may not offer a complete explanation.

A second hypothesis focuses on the importance of biotic factors—namely, predation—which may restrict arboreal species to nocturnality if predation pressure is high during the day in the canopy. Case studies have suggested that predators of Malagasy amphibians are primarily terrestrial snakes [32,63] and birds [64], which reflects findings across the tropics [65,66]. Arboreal amphibians may be especially vulnerable to bird predation during the day, when bird activity is highest. Bird predators tend to respond to visual and auditory cues including when foraging for amphibians [67], and male arboreal frogs conspicuously call at night while perched from branches or leaves. The predation avoidance strategy of remaining immobile, which is common for arboreal frogs [68], may be more effective at night than during the day, especially for the many bright green-coloured species of *Boophis* arboreal frogs in Madagascar. This contrasts with the morphology and behaviour of diurnally adapted Malagasy reptiles, such as notably cryptic chameleons [69]. Nocturnality has been posited as an anti-predation behaviour for mammals [70], and the same may apply to amphibians, contributing to why few arboreal amphibian species are diurnal worldwide [71]. Predation avoidance also may be a stronger driver of nocturnal–diurnal niche partitioning than food availability, at least for lizards and birds [72]. Thus, we propose that higher daytime than nighttime predation pressure may contribute to thermal niche underfilling by amphibians—especially for arboreal species. If so, the importance of biotic interactions for delineating the warm niche edge of species across vertitude (relative height within vegetated canopies) mirrors similar findings on the warm edges of altitudinal [60,73] and latitudinal [59] distributions of species. We encourage further investigation of biotic and abiotic drivers of vertical and diel niches, which may offer a convenient window into broader trends of biogeography.

Regardless of whether a hydric or biotic mechanism is at play, warm-end niche underfilling by nocturnal amphibians suggests they may have broad thermal safety margins, tolerating warmer conditions than their current exposure during activity [74]. This may suggest that nocturnal species, but perhaps not diurnal species, can withstand some climate warming—yet even some diurnal species may also be able to shift their diel niche to cooler nighttime conditions to avoid thermal stress [75]. However, we caution against interpreting such findings with undue optimism, as climate change may still negatively impact amphibians given drier and more variable climates, or reduction of key food sources and habitat [76].

### (c) Evolution, behaviour and morphology jointly impact thermal selection and thermoregulatory precision

We also found amphibians active in more thermally variable microclimates (namely, diurnal animals) had more narrow variation in Tsel than amphibians occurring in more thermally homogeneous conditions (electronic supplementary material, figure S4 and table S15). Substantial prior work has indicated that thermoregulatory behaviour is most effective when thermal heterogeneity exists in the environment [4,77,78]. Generally, organisms active within hotter and more thermally variable environments, such as desert lizards, face more severe consequences when body temperatures track external temperature (thermoconformity), and therefore must evolve precise abilities to track preferred temperatures (thermoregulation) [8,42]. Attempting to thermoregulate in thermally homogeneous environments, however, is more energetically costly as organisms must travel farther or spend more time seeking favourable temperatures [77,79]. Here we corroborate such theory and suggest that ectotherms adapted to activity in thermally homogeneous environments (in this case, nighttime conditions) have reduced opportunities for thermoregulation and therefore are poor behavioural thermoregulators: they have less fine-tuned abilities to identify and actively maintain preferred temperatures. Nocturnal animals may have less selective pressure to seek out favourable microclimates or avoid intolerably hot conditions, given the more homogeneous thermal environment they occupy when active. The higher thermal heterogeneity of daytime conditions increases both the capacity, and necessity, to thermoregulate for diurnally active animals [78]. Furthermore, during the daytime, nocturnal animals likely choose a resting location that is thermally buffered and within physiological limits. This passive experience of daytime temperatures by nocturnal animals further supports relaxed selection on the precision of their thermoregulatory behaviour.

Higher thermoregulatory precision by species active under thermally heterogeneous conditions, as we observed here, maybe due to an interplay of behaviour and evolution. As thermoregulatory behaviours buffer an organism from thermal variation, they maintain homeostasis and therefore limit exposure to environmental selection by thermal extremes. This slows the pace of physiological evolution, a phenomenon termed the ‘Bogert Effect’ [80,81]. Yet the ability to thermoregulate, and therefore the strength of the Bogert Effect, hinges upon the amount of microclimatic heterogeneity an organism is exposed to: the Bogert Effect may be stronger for diurnal species than nocturnal species [78,82]. Thus, the ‘evolutionary motor’ [77] driving selection for more precise thermoregulatory behaviour may be stronger for diurnal than nocturnal species, and such thermoregulation may in turn slow down physiological adaptation for diurnal species. We propose that vertical and diel niches may therefore jointly modulate the interaction of adaptation and behaviour for determining thermal traits, and encourage continued exploration of the interplay between the evolution of physiological traits and thermoregulatory behaviour.

We note that the thermoregulatory precision of an animal in a controlled experimental setting, as studied here, can differ from body temperatures in a natural setting, as organisms within more thermally variable environments tend to experience broader body temperatures [7]. Evidence is also mixed as to whether nocturnal animals may have different selected temperatures for nighttime activity and daytime rest [24,45,83]. Other mechanisms besides thermal heterogeneity may also impact an organism’s thermoregulatory precision, such as foraging style and breadth of dietary niche [79,83]. Given that our study was designed to focus on the mean selected temperature, rather than the range of selected temperatures, we see merit in future studies of how microhabitat fidelity and temporal activity influence the precision of thermoregulation.

We also found that there was a positive effect of an animal’s mass on its mean thermal selection, corroborating prior research on ectotherm Tsels [5,33,34]. Body size influences an organism’s heat budget by driving energy transfer from its environment, in which larger bodies have greater thermal inertia, changing temperature more slowly [34]. Furthermore, as larger ectotherms require more metabolic resources than smaller ectotherms, larger-bodied ectotherms require warmer temperatures to maintain metabolism [34]. Due to such mechanisms, Tsels tend to be positively correlated with body size [2,33], as we found here for rainforest amphibians. We note that while activity patterns may shape thermal selection, morphology will be consistently important given the fundamental biophysics of heat exchange and physiology.

## 5. Conclusion

Our findings of countergradient thermal selection by rainforest amphibians demonstrate divergence of behaviour in both space and time, with nocturnal amphibians in the trees selecting warmer temperatures than diurnal amphibians on the ground. Our study is the first, to our knowledge, that examines (and provides evidence for) divergent thermoregulatory behaviours for a speciose ectotherm community that occurs across the vertical forest gradient from floor to canopy. Adaptations for life aboveground may thus entail more than morphological and dispersal traits [23], but also physiological traits. We also found that amphibians underfill the warm end of their thermal niches—which we interpret as possibly the result of hydric and/or predatory constraints—and higher thermoregulatory precision of organisms active in thermally heterogeneous microclimates. We emphasize the importance of local spatial and temporal factors in delineating thermal physiology and behaviour [17]. Our results also open new lines of inquiry, such as the evolutionary underpinnings that result in disparate thermal selection, and how physiology and behaviour interact to influence vulnerability to global change. We encourage further work exploring the biogeographic axis of vertitude to understand community assembly and global change in forest environments, especially in the biodiverse tropics.

## Data Availability

Data are available via Zenodo [84]. Supplementary material is available online [85].
